# Correlation between PD-L1 expression and radiomic features in early-stage lung adenocarcinomas manifesting as ground-glass nodules

**DOI:** 10.3389/fonc.2022.986579

**Published:** 2022-09-13

**Authors:** Wenjia Shi, Zhen Yang, Minghui Zhu, Chenxi Zou, Jie Li, Zhixin Liang, Miaoyu Wang, Hang Yu, Bo Yang, Yulin Wang, Chunsun Li, Zirui Wang, Wei Zhao, Liang’an Chen

**Affiliations:** ^1^ Department of Respiratory and Critical Medicine, Medical School of Chinese People’s Liberation Army, Beijing, China; ^2^ Department of Respiratory and Critical Medicine, Chinese People’s Liberation Army General Hospital, Beijing, China; ^3^ Department of Pulmonary and Critical Care Medicine, Zhongnan Hospital of Wuhan University, Wuhan, China; ^4^ Department of Pathology, Chinese People’s Liberation Army General Hospital, Beijing, China; ^5^ Department of Thoracic Surgery, Chinese People’s Liberation Army General Hospital, Beijing, China; ^6^ Department of Radiology, Chinese People’s Liberation Army General Hospital, Beijing, China

**Keywords:** PD-L1, radiomics, prediction model, ground-glass nodules, lung adenocarcinoma

## Abstract

**Background:**

Immunotherapy might be a promising auxiliary or alternative systemic treatment for early-stage lung adenocarcinomas manifesting as ground-glass nodules (GGNs). This study intended to investigate the PD-L1 expression in these patients, and to explore the non-invasive prediction model of PD-L1 expression based on radiomics.

**Methods:**

We retrospectively analyzed the PD-L1 expression of patients with postoperative pathological diagnosis of lung adenocarcinomas and with imaging manifestation of GGNs, and divided patients into positive group and negative group according to whether PD-L1 expression ≥1%. Then, CT-based radiomic features were extracted semi-automatically, and feature dimensions were reduced by univariate analysis and LASSO in the randomly selected training cohort (70%). Finally, we used logistic regression algorithm to establish the radiomic models and the clinical-radiomic combined models for PD-L1 expression prediction, and evaluated the prediction efficiency of the models with the receiver operating characteristic (ROC) curves.

**Results:**

A total of 839 “GGN-like lung adenocarcinoma” patients were included, of which 226 (26.9%) showed positive PD-L1 expression. 779 radiomic features were extracted, and 9 of them were found to be highly corelated with PD-L1 expression. The area under the curve (AUC) values of the radiomic models were 0.653 and 0.583 in the training cohort and test cohort respectively. After adding clinically significant and statistically significant clinical features, the efficacy of the combined model was slightly improved, and the AUC values were 0.693 and 0.598 respectively.

**Conclusions:**

GGN-like lung adenocarcinoma had a fairly high positive PD-L1 expression rate. Radiomics was a hopeful noninvasive method for predicting PD-L1 expression, with better predictive efficacy in combination with clinical features.

## Introduction

Ground-glass nodules (GGNs) refer to the shadows with similar density of ground glass but not covering blood vessels and bronchial structures on computed tomography (CT) images. Lung cancer, tuberculosis, pneumonia and other lung diseases can all present such non-specific imaging manifestations. GGN bears the increased incidence with the popularization of low-dose CT screening and a significantly higher degree of malignancy than solid nodule, and the most common type of malignant GGN is early-stage lung adenocarcinoma (1). It’s reported that 95.5% stage 0/IA lung cancer patients detected by health screening were manifested as GGNs and 98.9% diagnosed as adenocarcinomas (2). Surgery is the main treatment strategy for early-stage lung cancer, whereas the 5-year recurrence-free survival (RFS) rate is about 85.5%-87.6% in these presented as mixed ground-glass nodules (mGGNs); in addition, treatment option for multifocal GGNs is limited in consideration of the nodule characteristics and the patient’s health condition, which lower the feasibility of curative surgery (3–5). Therefore, with a view to reduce recurrence risk in surgery patients and provide more treatment options for inoperable patients, “GGN-like lung adenocarcinoma” requires systemic therapy as an auxiliary or alternative to achieve individualized treatment.

Immune checkpoint inhibitors (ICIs) targeting programmed cell death-1/programmed cell death-ligand 1 (PD-1/PD-L1) pathway have been widely used in advanced non-small cell lung cancer (NSCLC) for the enduring efficacy and better safety, and the PD-L1 expression is approved by the Food and Drug Administration (FDA) as a predictive biomarker of ICI efficacy (6–8). Recently, several clinical trials have demonstrated that early-stage lung adenocarcinoma patients can benefit from neoadjuvant immunotherapy (9–14). Besides, studies also elucidated that PD-L1≥1% was positively associated with the major pathological response (MPR), pathological complete response (PCR), 3-year overall survival (OS) and disease-free survival (DFS) rates in neoadjuvant immunotherapy (15, 16). Therefore, PD-1/PD-L1 inhibitors are expected to be a promising remedy for early-stage lung adenocarcinoma patients and PD-L1 expression is likely to be the best predictive biomarker or one of the best biomarker combinations for predicting efficacy in these patients. Currently, PD-L1 expression in tumor tissues is mainly detected in surgical or biopsy specimens. Nevertheless, for early-stage lung adenocarcinoma patients who have not yet or cannot receive surgical treatment and whose biopsy specimens are difficult to obtain due to the size and location of GGNs, the PD-L1 detection is a bottleneck in the ICI treatment.

Radiomics means the process of manually or automatically extracting hundreds of quantitative features from medical images, reducing the dimensionality of the features, and ultimately using artificial intelligence methods such as machine learning or deep learning to build models relevant to clinical problems. Due to the panoramic analysis and dynamic monitoring of lesions, radiomics is widely used in precision medicine, especially in diagnosis, staging, treatment and prognosis of malignant tumors (17, 18). In the field of lung cancer, radiomics based on CT, positron emission tomography (PET)/CT and even magnetic resonance imaging (MRI) can not only contribute to the detection and identification of pulmonary nodules, the judgment of metastasis, the monitoring of treatment response and adverse events, and the assessment of prognosis, but also make some achievements in the prediction of mutated genes, immune microenvironment and even molecular markers (19–23). These studies indicated that microscopic changes at the histological and even molecular levels were correlated with macroscopic changes in imaging features. PD-1/PD-L1 pathway is an important immune checkpoint in lung adenocarcinoma, and abnormal expression of PD-L1 molecules would affect the tumor microenvironment and then change the overall morphology of the tumor, which can be captured by sophisticated imaging analysis. Several studies have focused on the noninvasive prediction of PD-L1 expression and have developed promising CT or PET/CT radiomic models; however, the sample size of these literatures was small, and most of the subjects were advanced NSCLC patients (24–26). So far, noninvasive prediction of PD-L1 expression in early-stage lung adenocarcinoma remains for further research. Considering chest CT is the most common clinical screening and follow-up tool for lung nodules, this study intends to analyze the PD-L1 expression in GGN-like lung adenocarcinomas, and explore the predicting impact of CT-based radiomic features on PD-L1 expression, thus facilitate the immunotherapy for these patients and contribute to precision medicine for early-stage lung adenocarcinoma.

## Materials and methods

### Patients

We retrospectively analyzed the clinicopathologic and CT imaging data of lung adenocarcinoma patients pathologically confirmed by resection surgery in the Department of Thoracic Surgery, Chinese PLA General Hospital from December 2018 to December 2020. Inclusion criteria: ①Chest CT examination is performed within 1 month before surgery, and the CT image of the lesion is presented as GGN; ②Postoperative pathological diagnosis is lung adenocarcinoma; ③PD-L1 test result of surgical specimen is available. Exclusion criteria: ①Solid nodule; ②Preoperative nonsurgical treatment of lung adenocarcinoma or needle biopsy was performed; ③CT image is unclear. A total of 839 patients were enrolled, including 280 males and 559 females. Their age ranged from 18 to 84 years, with an average of 55.7 ± 10.2 years. All patients were divided into negative group (n=613) and positive group (n=226) based on PD-L1 expression.

### PD-L1 expression

PD-L1 expression was detected on formalin-fixed paraffin-embedded surgical specimen, and immunohistochemical test was performed with PD-L1 IHC 22C3 pharmDx Kit (Dako Omnis) (Agilent Cat# GE00621-2, RRID: AB_2833074). PD-L1 expression level was measured by tumor proportion score (TPS) and positive PD-L1 expression was defined as TPS ≥ 1%.

### Clinicopathologic information

By searching medical records, the following information was collected: gender, age, body mass index (BMI), malignant tumor history, lung benign disease history (including chronic bronchitis, chronic obstructive pulmonary disease, bronchial asthma, tuberculosis), smoking history, family lung cancer history and other family malignant tumor history. Fasting venous blood was drawn within one week before operation for determination of white blood cell count (WBC), neutrophil percentage (NE), lymphocytes percentage (LP), carcinoembryonic antigen (CEA) and cytokeratin-19 fragment (CYFRA21-1), among which the white blood cell count with its categorical count was taken by the blood analyzer (SysmexXN9000, Sysmex), CEA and CYFRA21-1 level were determined by electrochemiluminescence (Roche, Cobas e602). According to the World Health Organization (WHO) Classification of Thoracic Neoplasms (Version 5, 2011), the pathological types of lung adenocarcinoma include precursor glandular lesions (PGL) and adenocarcinoma (AC). According to postoperative pathological results, none of the patients had lymph node infiltration, and their pathological staging was stage IA or IB.

### CT scanning protocols

Chest CT examination was performed on Brilliance iCT (Phillips Medical Systems). Scanning parameters: tube voltage 120kV, tube current 110mA, pitch 1, reconstruction layer thickness 1.00mm, layer spacing 1.00mm, reconstruction kernel iDose 3.

### Determination of CT morphological features

CT morphological features were evaluated by a radiologist with 5 years of experience and reviewed by a senior radiologist with 13 years of experience. Following features of GGNs were identified: ①type: pure groud-glass nodule (pGGN) and mGGN; ②diameter: the longest diameter of the nodule in the transverse maximum section; ③margin: clear or fuzzy; ④special signs: lobulation sign, spiculation sign, vacuole sign, pleural pull/indentation sign, vascular cluster sign, abnormal air bronchial sign.

### Extraction of radiomic and quantitative features

The entire workflow was done on the FDA-approved FACT Medical Imaging System (Dexin Medical Imaging Technology Company). Firstly, CT images in Digital Imaging and Communications in Medicine (DICOM) format were imported into the workstation, and images with thickness of 1.00mm were selected to enter the automatic processing mode of pulmonary nodules, including nodule recognition and region of interest (ROI) delineation. Then, under a fixed pulmonary window (window width 1500HU, window position -500HU), a respiratory physician with 5 years of clinical experience identified the target nodule and modified the ROI boundary layer by layer without knowing the patient’s pathological diagnosis. The principle of manual segmentation was that the ROI should cover as much of the nodule component area as possible and avoid surrounding vascular and bronchial structures. Next, a senior radiologist confirmed the ROI segmentation results of 50 randomly selected nodules. Eventually, the following parameters were automatically calculated and exported: 779 radiomic features (14 shape features, 24 first order features, 61 texture features and 680 wavelet features) and 15 quantitative features (volume, three-dimensional (3D) maximum diameter, 3D mean diameter, mean density, non-consolidation ratio, mass, surface area, pleural adhesion area, pleural proportion, fat proportion, surface area/volume (SA/V), calcification volume, mean vascular density, irregularity, and void volume ratio). In addition, we calculated inter-class correlation coefficient (ICC) to test the stability of radiomic features. The effect of different physician ROI segmentation levels on the stability of radiomic features was defined as inter-observer consistency, while the effect of ROI segmentation levels of the same physician at different time periods on the stability of radiomic features was defined as intra-observer consistency. Firstly, ROI segmentation was performed on 50 randomly selected CT images by WJS and MHZ; then the radiomic features were extracted for consistency analysis; lastly, radiomic features with ICC less than 0.75 were considered as inter-observer unstable features. After an interval of 1 month, WJS again performed ROI segmentation on previous 50 CT images, extracted radiomic features and performed consistency analysis. Radiomic features with ICC less than 0.75 were also considered as intra-observer unstable features.

### Statistical analysis and model building

**Figure 1:**
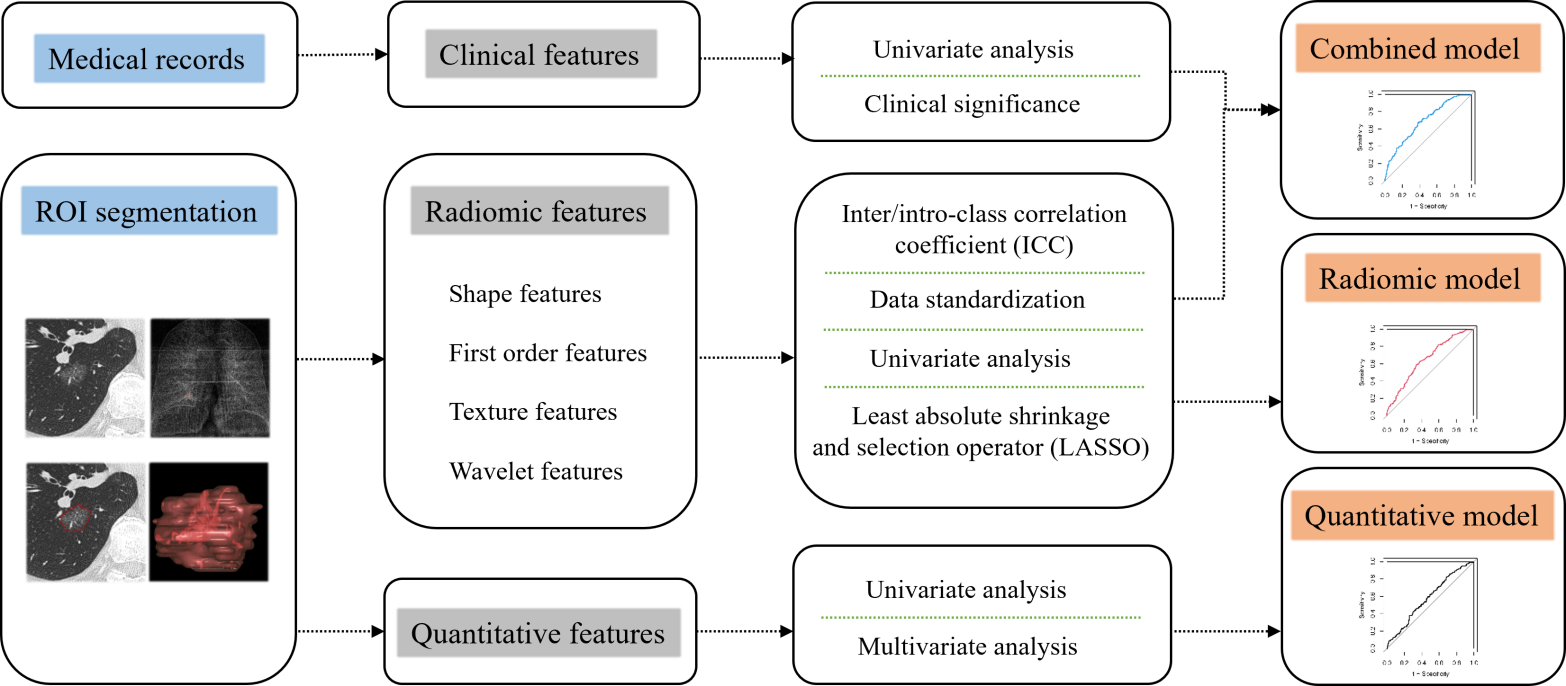
Flow charts of constructing radiomic model, combined model and quantitative model.

Data analysis was performed in IBM SPSS Statistics software (V26, RRID: SCR_016479). Continuous variables were compared using Mann-Whitney U test, and categorical variables using Chi-square or Fisher’s exact test. Missing values in laboratory results were supplemented by conditional mean completer. Variables with a P value less than 0.05 were considered statistically significant. R software (version 4.0.3) was used to build the model. The following R packages of psych, pROC, glmnet and e1071 were used during the establishment and evaluation process of models. The establishment process of radiomic model, clinical-radiomic combined model and quantitative model was shown in **Figure 1**. Radiomic features with ICC ≥ 0.75 were considered stable.

## Results

### Clinicopathologic characteristics of the patients

**Figure 2:**
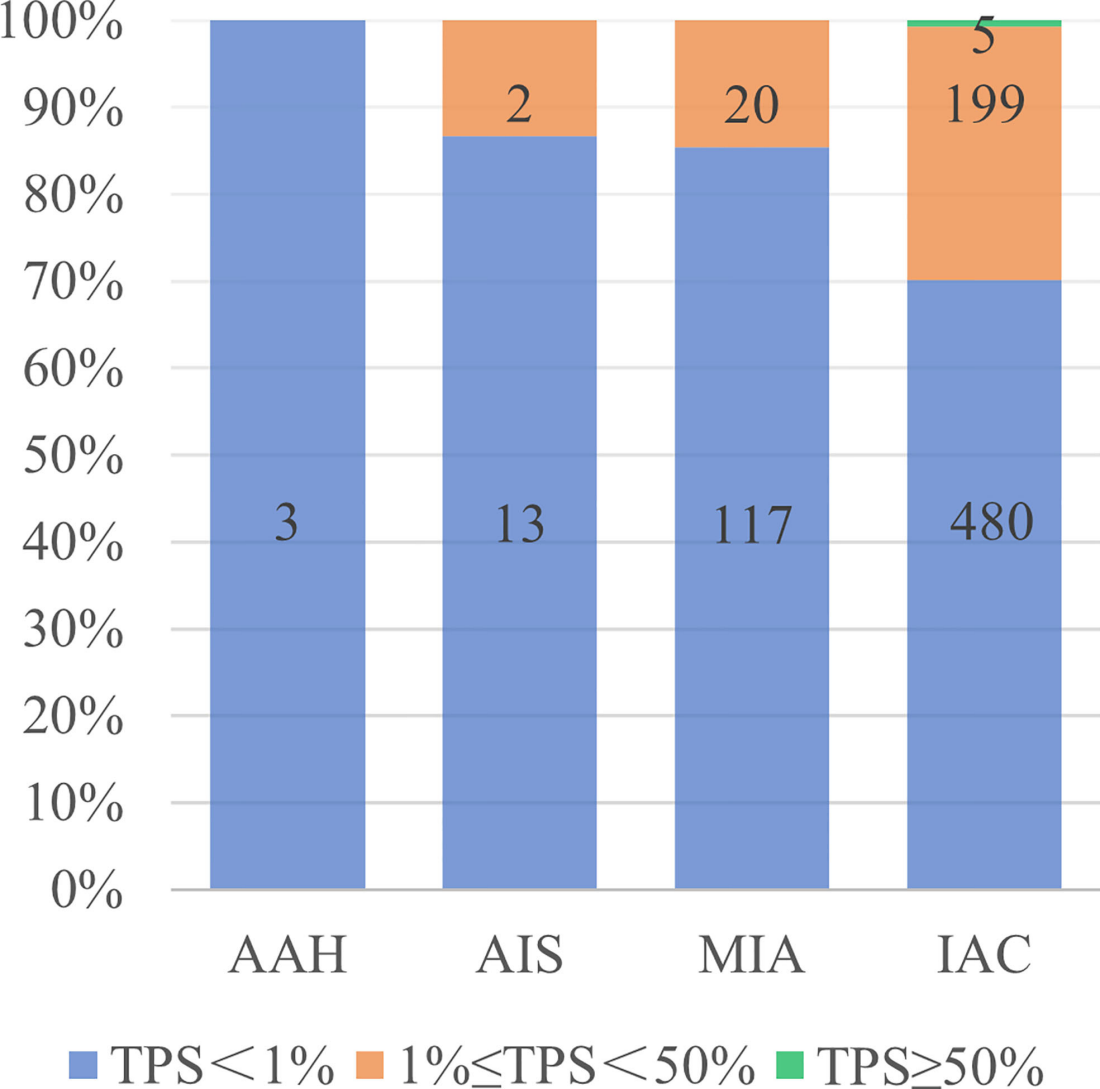
PD-L1 expression in different pathological types of lung adenocarcinoma. AAH, atypical adenomatous hyperplasia; AIS, adenocarcinoma in situ; MIA, microinvasive adenocarcinoma; IAC, invasive adenocarcinomas; TPS, tumor proportion score.

A total of 839 GGN-like lung adenocarcinoma patients were included, of which 226 (26.9%) patients showed positive PD-L1 expression, and 5 (0.6%) patients showed high PD-L1 expression (TPS≥50%). Positive PD-L1 expression rate showed an increasing trend from atypical adenomatous hyperplasia (AAH) to invasive adenocarcinoma (IAC), and all high PD-L1 expression patients were pathologically diagnosed as IACs (**Figure 2**). In the whole cohort, there was no correlation between PD-L1 expression and pathological type, but the positive PD-L1 expression rate in IB stage was higher than that in IA stage (P < 0.01). The age difference between positive group and negative group was statistically significant (P < 0.05), and patients in the positive group were older. The NE and CEA were positively correlated with PD-L1 expression, while the LP and CYFRA21-1 were negatively correlated with TPS. Other clinical characteristics, including gender, BMI, past history, family history and WBC, showed no statistical difference (**Table 1**). After randomization, there were statistically significant differences in gender, age, smoking history, pathological stage, CEA and CYFRA21-1 in the training cohort, while only CYFRA21-1 was associated with PD-L1 expression in the test cohort (**Table 2**).

**Table 1:** Comparison of clinicopathologic and CT morphological characteristics.

	Total	Negative group	Positive group	P value
		(TPS<1%)	(TPS≥1%)	
Gender				0.081
Male	280 (33.4)	194 (31.6)	86 (38.1)	
Female	559 (66.6)	419 (68.4)	140 (61.9)	
Age (years)	56.0 (49.0,63.0)	55.0 (49.0, 62.0.)	57.0 (51.0, 64.3)	**0.017**
BMI (kg/m^2^)	24.1(22.0,26.1)	24.1 (22.1, 26.1)	24.0 (22.0, 26.1)	0.871
Smoking history				0.108
Yes	142 (16.9)	96 (15.7)	46 (20.4)	
No	697 (83.1)	517 (84.3)	180 (79.6)	
Malignant tumor history				0.790
Yes	49 (5.8)	35 (5.7)	14 (6.2)	
No	790 (94.2)	578 (94.3)	212 (93.8)	
Lung benign disease history				0.286
Yes	41 (4.9)	27 (4.4)	14 (6.2)	
No	798 (95.1)	586 (95.6)	212 (93.8)	
Family lung cancer history				0.515
Yes	103 (12.3)	78 (12.7)	25 (11.1)	
No	736 (87.7)	535 (87.3)	201 (88.9)	
Family malignant tumor history				0.951
Yes	131 (15.6)	96 (15.7)	35 (15.5)	
No	708 (84.4)	517 (84.3)	191 (84.5)	
Pathological type				0.098
PGL	18 (2.1)	16 (2.6)	2 (0.9)	
AC	821 (97.9)	597 (97.4)	224 (99.1)	
Pathological stage				**0.003**
IA	167 (19.9)	107 (17.5)	60 (26.5)	
IB	672 (80.1)	506 (82.5)	166 (73.5)	
WBC (*10^9^)	5.55 (4.76, 6.21)	5.57 (4.76, 2.21)	5.55 (4.76, 6.10)	0.575
NE	0.556 (0.514, 0.609)	0.556 (0.514, 0.605)	0.567 (0.516, 0.623)	**0.043**
LP	0.347 (0.297, 0.390)	0.347 (0.301, 0.390)	0.335 (0.288, 0.390)	**0.010**
CEA (ug/L)	1.89 (1.43, 2.18)	1.89 (1.39, 1.97)	2.18 (1.55, 2.25)	**<0.001**
CYFRA21-1 (ng/mL)	2.50 (2.03, 2.50)	2.50 (2.07, 2.53)	2.37 (1.94, 2.37)	**<0.001**
Nodule type				**0.005**
pGGN	489(58.3)	375(61.2)	114(50.4)	
mGGN	350(41.7)	238(38.8)	112(49.6)	
Diameter (mm)	13.0(9.7,18.0)	12.0(9.0, 17.5)	14.0(10.0, 18.0)	**0.003**
Margin				0.188
Clear	791(94.3)	574(93.6)	217(96.0)	
Fuzzy	48(5.7)	39(6.4)	9(4.0)	
Lobulation sign				0.745
Yes	227(27.1)	164(26.8)	63(27.9)	
No	612(72.9)	449(73.2)	163(72.1)	
Spiculation sign				0.566
Yes	121(14.4)	91(14.8)	30(13.3)	
No	718(85.8)	522(85.2)	196(86.7)	
Vacuole sign				0.150
Yes	107(12.8)	72(11.7)	35(15.5)	
No	732(87.2)	541(88.3)	191(84.5)	
Pleural pull/indentation sign				0.257
Yes	143(17.0)	99(16.2)	44(19.5)	
No	696(83.0)	514(83.4)	182(80.5)	
Vascular cluster sign				0.361
Yes	127(15.1)	97(15.8)	30(13.3)	
No	712(84.9)	516(84.2)	196(86.7)	
Abnormal air bronchial sign				0.363
Yes	87(10.4)	60(9.8)	27(11.9)	
No	752(89.6)	553(90.2)	199(88.1)	

TPS, tumor proportion score; BMI, body mass index; PGL, precursor glandular lesions; AC, adenocarcinoma; WBC, white blood cell count; NE, neutrophil percentage; LP, lymphocytes percentage; CEA, carcinoembryonic antigen; CYFRA21-1, cytokeratin-19 fragment; pGGN, pure ground-glass nodule; mGGN, mixed ground-glass nodule.

Bold values indicate that the P-value of this feature is less than 0.05, which is statistically significant.

**Table 2:** Comparison of clinicopathologic features in training cohort and test cohort.

	Training cohort			Test cohort		
	Negative group	Positive group	P value	Negative group	Positive group	P value
Total	429	158		184	68	
Gender			**0.027**			0.870
Male	127 (29.6)	62 (39.2)		67 (36.4)	24 (35.3)	
Female	302 (70.4)	96 (60.8)		117 (63.6)	44 (64.7)	
Age (years)	55.0 (49.0, 62.0)	58.0 (51.0, 65.0)	**0.003**	57.0 (51.0, 63.0)	55.5 (50.0, 64.0)	0.885
BMI (kg/m^2^)	23.9 (22.0, 26.0)	24.2 (22.3, 26.3)	0.295	24.5 (22.3, 26.2)	23.6 (21.8, 26.0)	0.203
Smoking history			**0.016**			0.639
Yes	53 (12.4)	32 (20.3)		43 (23.4)	14 (20.6)	
No	376 (87.6)	126 (79.7)		141 (76.6)	54 (79.4)	
Malignant tumor history			0.785			1.000
Yes	22 (5.1)	9 (5.7)		13 (7.1)	5 (7.4)	
No	407 (94.9)	149 (94.3)		171 (92.9)	63 (92.6)	
Lung benign disease history			0.328			0.706
Yes	21 (4.9)	11 (7.0)		6 (3.3)	3 (4.4)	
No	408 (95.1)	147 (93.0)		178 (96.7)	65 (95.6)	
Family lung cancer history			0.914			0.307
Yes	53 (12.4)	19 (12.0)		25 (13.6)	6 (8.8)	
No	376 (87.6)	139 (88.0)		159 (86.4)	62 (91.2)	
Family tumor history			0.885			0.908
Yes	70 (16.3)	25 (15.8)		26 (14.1)	10 (14.7)	
No	359 (83.7)	133 (84.2)		158 (85.9)	58 (85.3)	
Pathological type			0.529			0.195
PGL	10 (2.3)	2 (1.3)		6 (3.3)	0 (0.0)	
AC	419 (97.7)	156 (98.7)		178 (96.7)	68 (100.0)	
Pathological stage			**0.007**			0.227
IA	353 (82.3)	114 (72.2)		153 (83.2)	52 (76.5)	
IB	76 (17.7)	44 (27.8)		31 (16.8)	16 (23.5)0	
WBC (*10^9^)	5.44 (4.75, 6.11)	5.44 (4.70, 6.10)	0.899	5.44 (4.82, 6.35)	5.44 (4.80, 6.22)	0.665
NE	0.557 (0.510, 0.601)	0.557 (0.513, 0.612)	0.277	0.557 (0.517, 0.617)	0.565 (0.533, 0.636)	0.313
LP	0.342 (0.306, 0.394)	0.342 (0.294, 0.391)	0.089	0.342 (0.294, 0.388)	0.336 (0.285, 0.364)	0.450
CEA (ug/L)	1.89 (1.36, 1.89)	2.18 (1.57, 2.40)	**<0.001**	1.89 (1.41, 2.11)	2.13 (1.51, 2.18)	0.067
CYFRA21-1 (ng/mL)	2.50 (2.09, 2.50)	2.37 (2.00, 2.44)	**<0.001**	2.50 (2.04, 2.71)	2.37 (1.82, 2.37)	**<0.001**

BMI, body mass index; PGL, precursor glandular lesions; AC, adenocarcinoma; WBC, white blood cell count; NE, neutrophil percentage; LP, lymphocytes percentage; CEA, carcinoembryonic antigen; CYFRA21-1, cytokeratin-19 fragment.

Bold values indicate that the P-value of this feature is less than 0.05, which is statistically significant.

### CT morphological features of the patients

The difference in nodule type between positive group and negative group was statistically significant, and the proportion of mGGN was larger in positive group (P < 0.01). The longer nodule diameter was correlated with the positive PD-L1 expression (P < 0.01). There were no statistically significant differences in nodule margin, peripheral features and internal features between the two groups (**Table 1**).

### Radiomic model

**Figure 3:**
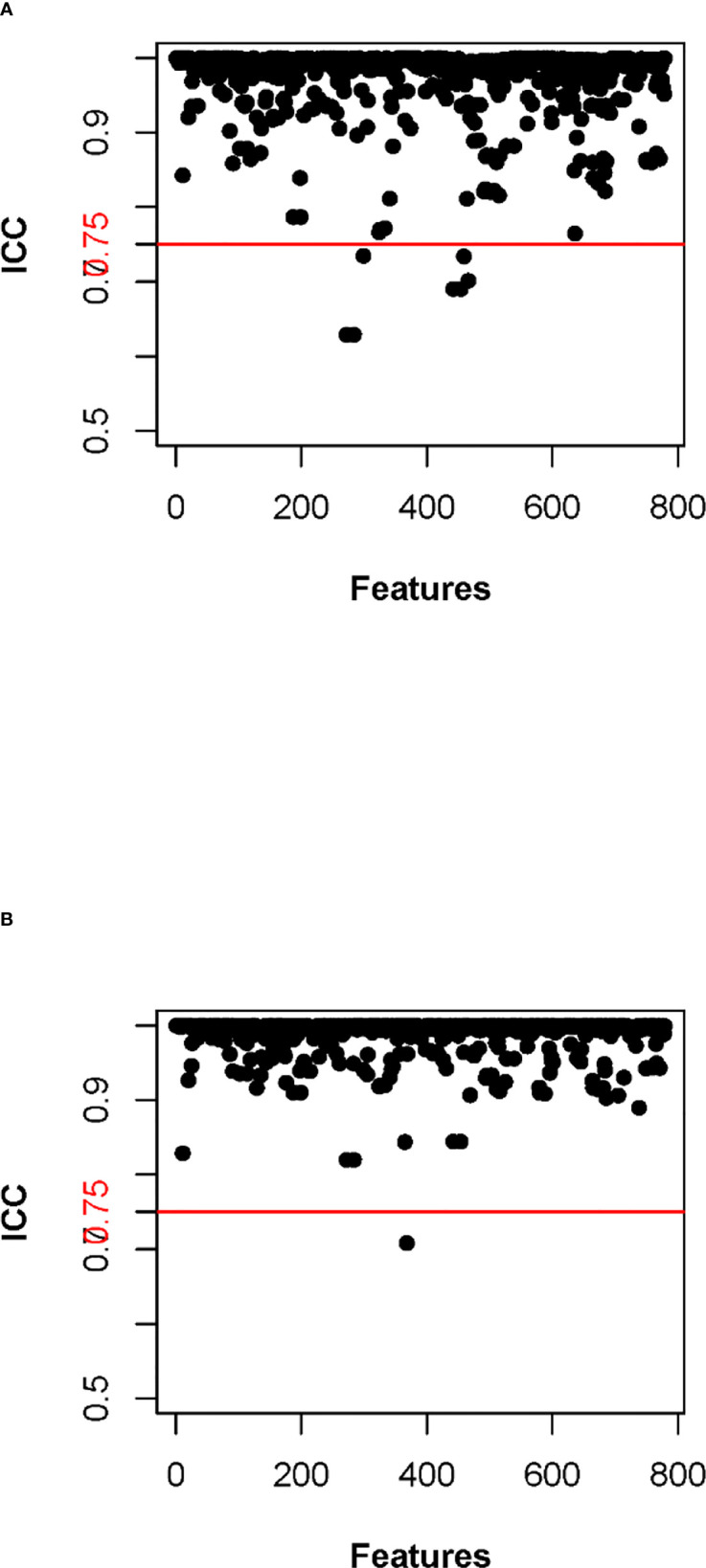
Inter-class correlation coefficient (ICC) of inter-observers **(A)** and intra-observer **(B)**.

**Figure 4:**
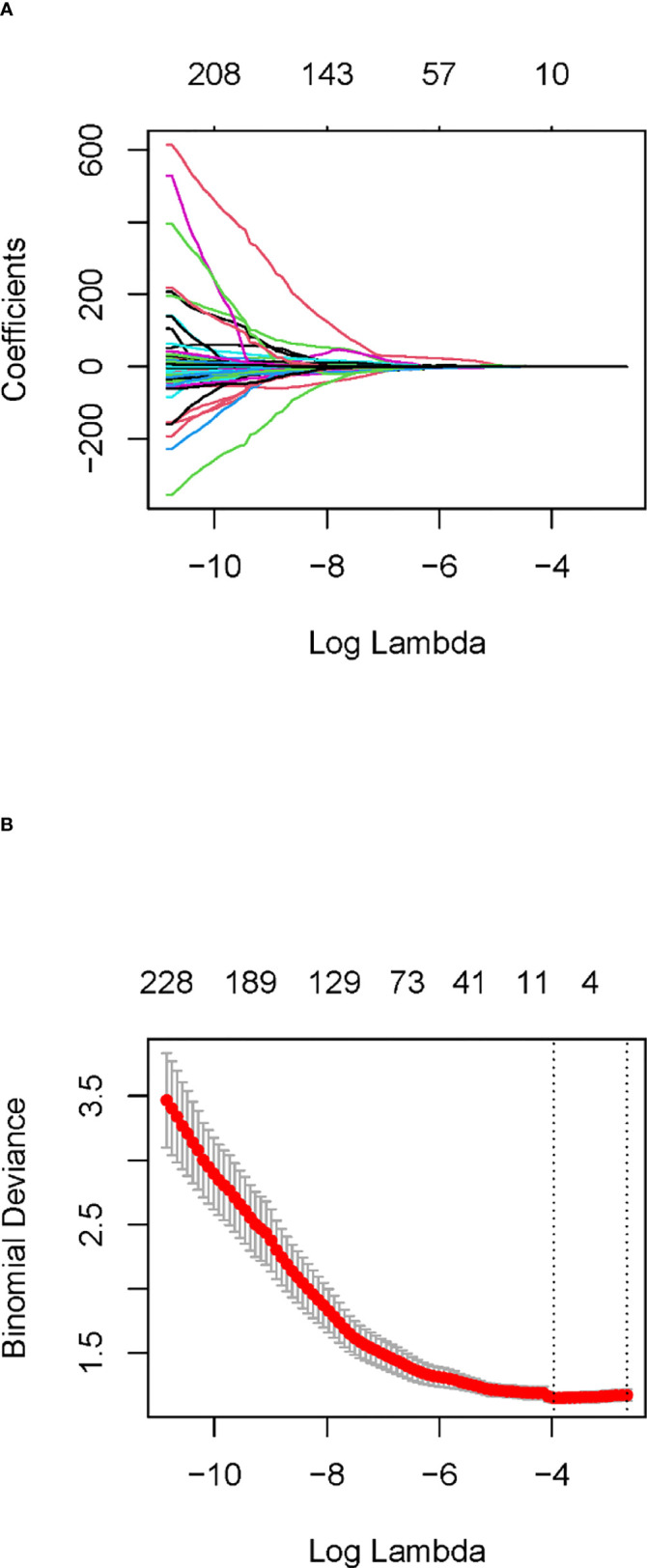
Least absolute shrinkage and selection operator (LASSO) coefficient profiles **(A)** and determining the parameter Lambda (λ) in the LASSO model with 10-fold cross-validation **(B)**.

**Figure 5:**
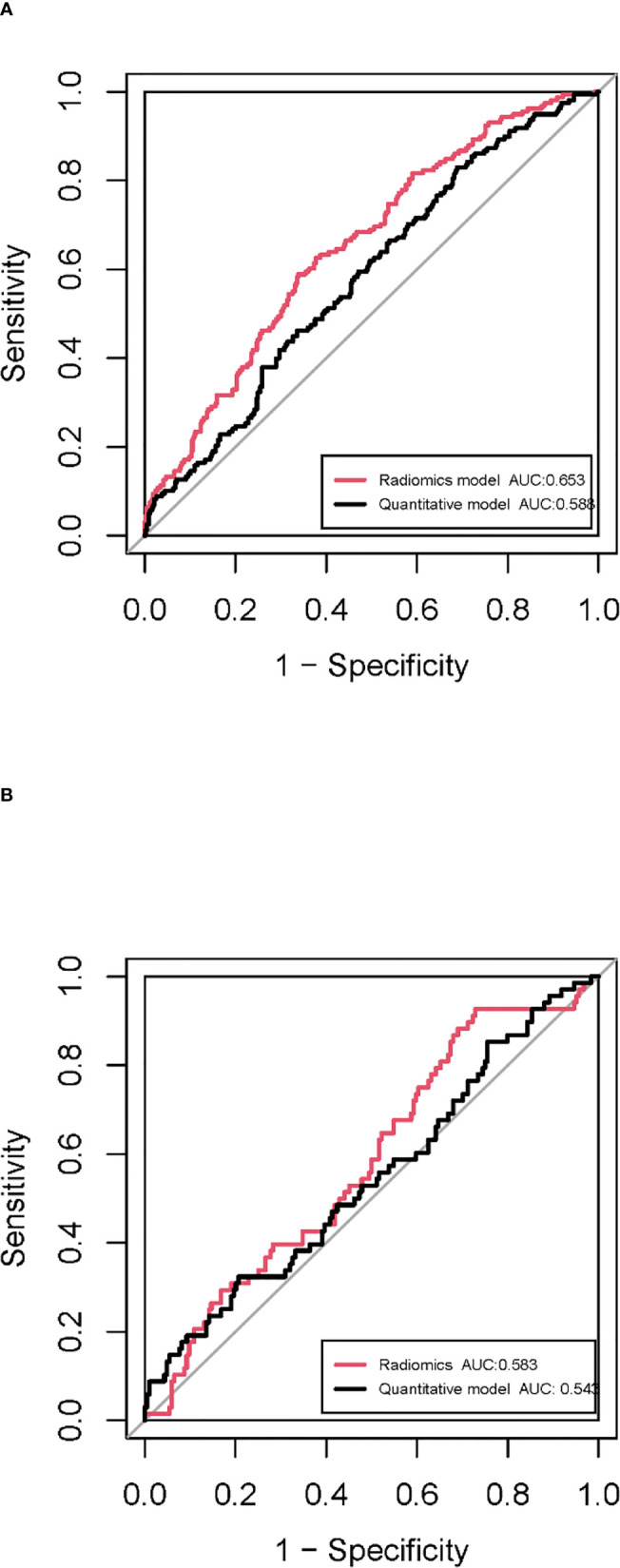
ROC curves of the radiomic model and quantitative model in the training cohort **(A)** and the test cohort **(B)**.

**Figure 6:**
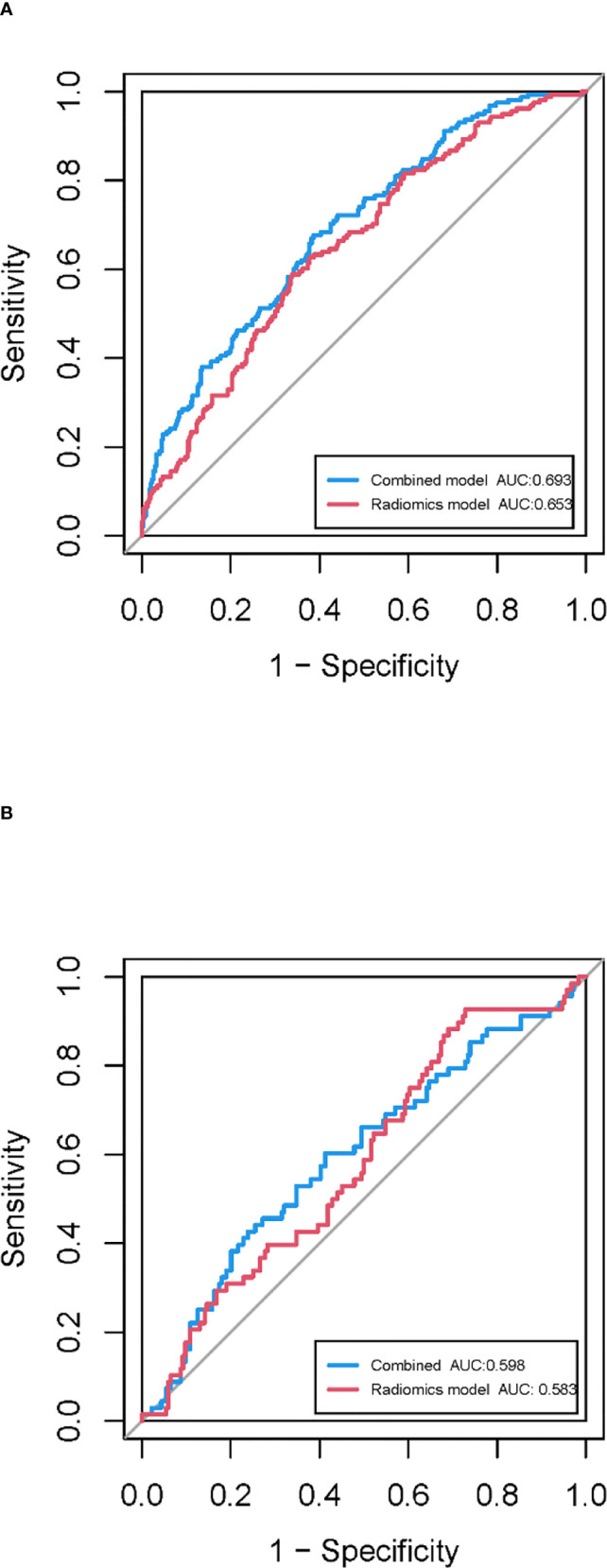
ROC curves of the combined model and radiomic model in the training cohort **(A)** and the test cohort **(B)**.

In the training cohort, 762 radiomics features were stable (ICC ≥ 0.75) both inter- intra- observer (**Figure 3**). 301 features were statistically significant in univariate analysis (P < 0.05). 9 features (**Table 3**) were eventually selected by least absolute shrinkage and selection operator (LASSO) to establish radiomic model (**Figure 4**). The area under the curve (AUC) values of the radiomic models in the training cohort and the test cohort were 0.653 and 0.583 respectively (**Figures 5**, **6**), and the negative predictive values were 81.4% and 75.7%, the positive predictive values were 39.1% and 30.2%, the accuracies were 57.4% and 54.8%, the sensitivities were 58.9% and 51.5%, the specificities were 66.2% and 56% in the training cohort and the test cohort.

**Table 3:** Selected radiomic features in training cohort.

Number			
1	0riginal	firstorder	75 Percentile
2	0riginal	Shape	Maximum 2D Diameter Slice
3	0riginal	glcm	Correlation
4	0riginal	glszm	Large Area High Gray Level Emphasis
5	wavelet-LHH	glcm	Maximal correlation coefficient
6	wavelet-LHH	ngtdm	Busyness
7	wavelet-HLL	glrlm	Long Run Low Gray Level Emphasis
8	wavelet-LLL	firstorder	Interquartile Range
9	wavelet-LLL	glszm	Large Area Low Gray Level Emphasis

### Quantitative model

In the training cohort, univariate and multivariate analyses identified that SA/V and irregularity were independent risk factors for PD-L1 expression. Smaller SA/V and greater irregularity were correlated with positive PD-L1 expression (P < 0.05) (**Table 4**). The AUC values of the quantitative models were 0.588 and 0.545 in the training cohort and the test cohort respectively (**Figure 5**).

**Table 4:** Data analysis of quantitative features in training cohort.

	univariate analysis			multivariate analysis		
	Negative group	Positive group	P value	P value	β	OR (95%CI)
	(TPS<1%)	(TPS≥1%)				
Volume (ml)	0.88 (0.42, 2.31)	1.31 (0.61, 2.94)	**0.001**	0.419		
3D maximum diameter(mm)	1.60 (1.19, 2.32)	1.93 (1.30, 2.43)	**0.005**	0.268		
3D mean diameter(mm)	1.16 (0.91, 1.62)	1.30 (1.04, 1.72)	**0.003**	0.764		
Mean density (HU)	566.57 (480.53, 641.61)	541.19 (443.40, 637.90)	0.101			
Non-consolidation ratio	0.37 (0.24, 0.51)	0.36 (0.22, 0.54)	0.815			
Mass(g)	0.21 (0.09, 0.55)	0.33 (0.13, 0.74)	**0.003**	0.507		
Surface area(cm^2^)	5.56 (3.20, 12.28)	7.76 (4.05, 14.19)	**0.002**	0.276		
Pleural adhesion area (cm^2^)	0.27 (0.13, 0.60)	0.37 (0.17, 0.79)	**0.016**	0.796		
Pleural proportion	0.05 (0.04, 0.07)	0.05 (0.04, 0.07)	0.947			
Fat proportion	0.02 (0.01, 0.04)	0.02 (0.01, 0.05)	0.051			
Surface area/volume ratio	6.36 (5.22, 7.58)	5.87 (4.90, 7.07)	**0.002**	**0.009**	-0.348	0.706 (0.544, 0.917)
Calcification volume(mm^3^)	14.65 (4.27, 32.09)	16.66 6.83, 44.98)	**0.001**	0.078		
Mean vascular density (HU)	0.20 (0.09, 0.53)	0.30 (0.13, 0.72)	**0.002**	0.508		
Irregularity	0.58 (0.34, 0.82)	0.68 (0.42, 0.92)	**0.008**	**0.015**	1.864	6.449 (1.438, 28.915)
Void volume ratio	0.09 (0.05, 0.14)	0.09 (0.05, 0.16)	0.395			

TPS, tumor proportion score; 3D, three-dimensional.

Bold values indicate that the P-value of this feature is less than 0.05, which is statistically significant.

### Combined model

The clinical-radiomic combined model was constructed from both 9 radiomic features and 7 clinical features, the latter including statistically significant characteristics (age, NE, LP, CEA and CYFRA21-1) in the whole cohort and clinically significant characteristics (sex and smoking history). the AUC values of the combined models were 0.693 and 0.598 in the training cohort and the test cohort respectively (**Figure 6**), and the negative predictive values were 83.8% and 79.8%, the positive predictive values were 39.2% and 32.6%, the accuracies were 63.0% and 54.0%, the sensitivities were 67.7% and 66.2%, the specificities were 61.3% and 49.5% in the training cohort and the test cohort.

## Discussions

Our study unveiled the quite PD-L1 expression status in GGN-like lung adenocarcinomas, and demonstrated that the CT-based radiomic model could distinguish between negative and positive PD-L1 expression.

Though immunotherapy has the advantages of lasting efficacy and less serious adverse events, the spectrum of patients suitable for ICI treatment is narrow, with only about 20% overall radiological response (ORR) rate in advanced-stage lung cancer (27). MPR of neoadjuvant immunotherapy was also dissatisfactory, ranging from 17% to 31% (28).

PD-L1 is the key molecule of immune checkpoint pathway, making it a predictive biomarker for ICI treatment (29), and positive PD-L1 expression is associated with higher ICI response rates in both advanced and early lung cancer patients (30–32). Thus, exploring the PD-L1 expression in GGN-like lung adenocarcinoma is essential for confirming the feasibility of immunotherapy and identifying eligible patients.

This study indicated that in GGN-like lung adenocarcinomas, the positive PD-L1 expression rate was about 26.9%, while those with high PD-L1 expression were rare. Previous studies also investigated PD-L1 expression in surgical specimen of NSCLC. Pan et al. discovered that the positive PD-L1 patients accounted for only 4.1% in Chinese lung adenocarcinoma patients, which might be attributed to their grade criteria of PD-L1 expression (There are 0~3+ grades, where 0 and 1+ means negative) to a large extent (33). A Japanese study showed that the positive PD-L1 rate was 21.9%, and the PD-L1 expression in adenocarcinoma *in situ* (AIS), microinvasive adenocarcinoma (MIA) and lepidic predominant adenocarcinoma (LPA) was all negative (34). In our study, a larger sample size was included and more optimistic PD-L1 expression results were obtained. The positive PD-L1 rate in PGL was 11.1%, and that in AC was 27.3%. Another European study observed that the positive PD-L1 rate was 30.8%, and the high PD-L1 expression rate was 10.4%. Meanwhile, it uncovered that the PD-L1 expression was correlated with gender and smoking history, and was highly correlated with tumor grade and lymph node invasion (35). In contrast, more GGN-like lung adenocarcinoma patients in our study were female and non-smokers, and all patients had no lymph node infiltration, which may explain the lower positive PD-L1 rate. In general, the positive PD-L1 rate of GGN-like lung adenocarcinomas was comparable to other early-stage lung adenocarcinomas, confirming that immune escape mechanism acted as a super-early event in cancer development (36), thus laying the foundation for the application of ICIs. Immunity, inflammation and their interactions play an important role in the occurrence, development and progression of cancer (37, 38). Studies have shown that both neutrophil-to-lymphocyte ratio (NLR) and absolute lymphocyte count (ALC) can predict the prognosis of nivolumab-treated NSCLC patients (39, 40). Our study also proved that higher NE and lower LP were associated with positive PD-L1 expression. Moreover, we discovered that higher baseline CEA level and lower baseline CYFRA21-1 level might be potential markers of PD-L1 expression, whereas other studies had come to conflicting conclusions (41, 42). The relationship between tumor marker levels and PD-L1 expression in early-stage lung adenocarcinomas has not been extensively studied yet, and more data are needed for further confirmation. Univariate analysis of clinical characteristics in the whole cohort and the training cohort showed that gender, age, smoking history, NE, LP, CEA and CYFRA21-1 might be associated with the PD-L1 expression, which was also recognized in previous studies. Therefore, above clinical features were added into the clinical-radiomic combined model. It can be seen from **Table 2** that few clinical features were statistically different in the test cohort, resulting in a modest improvement in the AUC value of the combined model, which suggested that the correlation between clinical features and PD-L1 expression in GGN-like lung adenocarcinoma needed to be further verified.

PD-L1 expression was associated with nodule type and diameter in this study. In addition to these two CT morphological features, other studies also found that irregular shape, pleural indentation sign, air bronchogram sign, the convergence sign and cavitation sign were correlated with PD-L1 expression (43, 44). These associations of radiological features with PD-L1 expression suggested that pathological and even molecular discrepancy in tumors can be externalized in imaging. The lack of significant CT morphological features in this study might be attributed to the fact that TPS=5% was used as a threshold in above two studies, magnifying the radiological feature difference between groups. Since PD-L1 expression level in early-stage lung adenocarcinoma was relatively low and there were only 9.3% (78/839) GGNs with TPS≥5% in this study, it is statistically difficult to further explore the relationship between CT morphological features and PD-L1 expression by increasing the cut-off value. On the other hand, due to the limitation of volume and density, the internal and peripheral features of GGN were not clear enough to be accurately distinguished by naked eyes. Hence, further evaluation of radiological features in GGN-like lung adenocarcinoma is conducive to the evaluation of PD-L1 expression.

Quantitative CT objectively and quantitatively depict the size, shape and special signs of nodules, which is more dependable than clinician’s determination; it has been applied in the diagnosis, evaluation and prediction of lung diseases (45–47). As shown in **Table 4**, most quantitative features were correlated with the PD-L1 expression in the training cohort, nevertheless, the quantitative model established by SA/V and irregularity had poor prediction efficiency for PD-L1 expression. It can be concluded that the image information extracted by mere quantitative CT is limited.

Radiomics extracts massive quantitative features from different angles of original images and transformed images, excavating as much image information as possible, thus is more effective than quantitative CT. At present, radiomic method has been fully developed, and its application in lung cancer has progressed from qualitative diagnosis and histological identification to the present level of gene or molecular detection (48–51). Recent studies on noninvasive prediction of PD-L1 expression mainly focus on advanced NSCLC, and the PET/CT-based radiomic model has achieved good prediction effect (52). Due to the low sensitivity of PET/CT in the diagnosis of GGN-like lung adenocarcinomas, thin-layer CT is mostly used for the detection and follow-up of pulmonary nodules. We established a CT-based radiomic model in GGN-like lung adenocarcinoma and found that it had the potential of noninvasive prediction for PD-L1 expression (AUC were 0.653 versus 0.583 in training cohort and test cohort). Several studies have also attempted to establish CT-based PD-L1 expression prediction models in advanced NSCLC, but yielded inconsistent results. Bracci et al. created two radiomic models based on 48 texture features: one model determining whether TPS ≥1% achieved AUC values of 0.763 and 0.806 in the training cohort (n=48) and the validation cohort (n=24), and the other for TPS≥50% got AUC values of 0.811 and 0.789 respectively (25). Sun et al. built a radiomic model for PD-L1 expression≥50% on a much larger data set (390 patients, 200 texture features), and achieved the similar predictive effect (AUC: 0786 and 0.807) (42). The above two favorable results might be attributed to the higher PD-L1 expression level in advanced NSCLC, and the greater differentiation between groups with the TPS cut-off of 50%. However, another study did not seem to support this hypothesis: Yoon et al. extracted 58 radiomic features in 153 advanced adenocarcinoma patients, established a clinical-radiomic combined model for TPS≥50%, and came up with an AUC of 0.667 (24). In fact, the proportion of squamous cell carcinoma in the positive group was higher in the first two studies; since positive PD-L1 expression rate in lung squamous cell carcinoma was significantly higher than that in lung adenocarcinoma (53), better discrimination ability of models could be partly explained by differences in histological types between groups. In addition, due to the smaller sample size and radiomic feature number of the above three studies, the robustness of the models needs to be further verified. Our study focused on the noninvasive prediction of PD-L1 expression in early-stage GGN-like lung adenocarcinoma, and obtained meaningful radiomic models. Firstly, the radiomic features showed good robustness, and 97.8% of them reached the preset ICC. A study revealed that the accuracy of lesion segmentation was less affected by the training level and clinical experience of physicians (54). It might be concluded that regular shape and clear boundary of most GGNs facilitated the ROI segmentation process. Secondly, it is reported that wavelet features have better repeatability and reproducibility (55). Our study covered 680 wavelet features, and over half radiomic features ultimately applied to the prediction model were wavelet features. Thirdly, neither the single radiomic model nor the clinical model had better predictive efficacy than the combined model (24, 56–58), indicating that the combination of radiomic and clinical features was more conducive to accurate prediction of PD-L1 expression. Our study also confirmed that the clinical-radiomic combined model was indeed superior to the radiomic model. Despite this, the prediction performance of either radiomic model or combined model remained not ideal and the discrimination error of positive PD-L1 expression were large. The lower PD-L1 expression level in GGN-like lung adenocarcinoma patients and more patients with negative PD-L1 expression included in this study might be one of the reasons. Besides, 1% was used as the cutoff value of PD-L1 expression in this paper, which also resulted in the imperceptible image difference between the negative group and the positive group. Furthermore, the radiomic features included in our and other PD-L1 expression prediction models were different from each other, and these studies lacked external validation data. Though the traditional radiomics method has a relatively mature operation process, there is poor consistency among different studies due to the diversity of original medical image protocols, image segmentation platforms, radiomic feature categories and machine learning algorithms. so we need to expand the patient population and adopt more advanced radiomic methods such as convolutional neural networks to achieve the clinical application of PD-L1 non-invasive prediction. Also, since the predictive efficacy of a single biomarker is limited, PD-L1 could combine genomics, proteomics and so on to construct multi-omics biomarkers.

Our study had several limitations: First, this was a single-center retrospective study with intrinsic bias, patients undergoing thoracic surgery in the hospital were not generally representative and the prediction model was not externally validated; Second, the semi-automatic ROI segmentation was performed by respiratory physicians, though the radiologist confirmed the results of 50 randomly selected nodules the objectivity and reproducibility of the radiomic features still needed to be further verified; Third, the absence of follow-up and prognostic assessment of patients made it impossible to establish an association between radiomic and recurrence or survival for improving the individualized treatment of early-stage lung adenocarcinoma.

In conclusion, early-stage lung adenocarcinomas manifesting as GGNs had a fairly high positive PD-L1 expression rate. Due to the advantages of noninvasiveness and repeatability, the radiomic-based model could better predict the PD-L1 expression of GGNs, thus paves the way of a more accurate diagnosis and treatment scheme for the individualized treatment of early-stage GGN-like lung adenocarcinoma.

## Data availability statement

The raw data supporting the conclusions of this article will be made available by the authors, without undue reservation.

## Ethics statement

The studies involving human participants were reviewed and approved by the Medical Ethics Committee of the Chinese People’s Liberation Army General Hospital. The patients/participants provided their written informed consent to participate in this study.

## Author contributions

LC and WZ designed the study. WS and ZY reviewed literature, interpreted the data and wrote manuscripts. WS, MZ, JL, MW, HY and CZ collected data. WS, MZ, YW, BY and ZW segmented the ROI. ZY, MZ, CZ and ZL processed the data and analyzed the statistics. LC, WZ, JL and CL revised the manuscript. All authors contributed to the article and approved the submitted version.

## Funding

This study was supported by Beijing Capital Development Special Project for Health Research [2020-1-5100] and Big Data Project of Chinese People’s Liberation Army General Hospital [2019MBD-052].

## Conflict of interest

The authors declare that the research was conducted in the absence of any commercial or financial relationships that could be construed as a potential conflict of interest.

## Publisher’s note

All claims expressed in this article are solely those of the authors and do not necessarily represent those of their affiliated organizations, or those of the publisher, the editors and the reviewers. Any product that may be evaluated in this article, or claim that may be made by its manufacturer, is not guaranteed or endorsed by the publisher.

## Glossary

**Table:**

GGN	Ground-glass nodule
CT	computed tomography
RFS	recurrence-free survival
mGGN	mixed ground-glass nodule
ICI	immune checkpoint inhibitor
PD-1/PD-L1	programmed cell death-1/programmed cell death-ligand 1
NSCLC	non-small cell lung cancer
FDA	Food and Drug Administration
MPR	major pathological response
PCR	pathological complete response
OS	overall survival
DFS	disease-free survival
PET	positron emission tomography
MRI	magnetic resonance imaging
TPS	tumor proportion score
BMI	body mass index
WBC	white blood cell count
CEA	carcinoembryonic antigen
CYFRA21-1	cytokeratin-19 fragment
WHO	World Health Organization
PGL	precursor glandular lesions
AC	adenocarcinoma
pGGN	pure groud-glass nodule
DICOM	Digital Imaging and Communications in Medicine
ROI	region of interest
3D	three-dimensional
SA/V	surface area/volume
ICC	inter-class correlation coefficient
AAH	atypical adenomatous hyperplasia
AIS	adenocarcinoma <italic>in situ</italic>
MIA	microinvasive adenocarcinoma
IAC	invasive adenocarcinomas
NE	neutrophil percentage
LP	lymphocytes percentage
LASSO	least absolute shrinkage and selection operator
AUC	Area under the curve
NLR	neutrophil-to-lymphocyte ratio
ALC	absolute lymphocyte count
ORR	overall radiological response
LPA	lepidic predominant adenocarcinoma
ROC	receiver operating characteristic
